# A qualitative investigation into the psychological experiences of patients undergoing thoracotomy for the removal of intraesophageal fishbone

**DOI:** 10.1186/s12876-025-04421-w

**Published:** 2025-11-18

**Authors:** Xiaoying Hou, Junhui Wang, Wenbin Zou

**Affiliations:** Department of Thoracic Surgery, Tongji Hospital, Tongji Medical College, Huazhong University of Science and Technology, 1095 Jiefang Avenue, Qiaokou District, Wuhan, Hubei Province, Wuhan, 430030 China

**Keywords:** Esophageal foreign body, Fish bone, Psychological experience, Qualitative research

## Abstract

**Background:**

Esophageal foreign bodies, especially fish bones, are common emergencies that may require surgery like thoracotomy. Psychological impacts such as pain and anxiety are often overlooked. This study explores patients’ psychological experiences during diagnosis, treatment, and recovery to inform effective interventions and improve outcomes.

**Methods:**

Between May 2022 and May 2023, we employed a purposive sampling technique to select 12 patients who had undergone thoracotomy for the removal of intraesophageal fishbone as participants in our study. Utilizing a phenomenological research framework, we conducted semi-structured interviews and applied Colaizzi’s seven-step method for data analysis to identify emergent themes.

**Results:**

The psychological experiences of patients at different stages of thoracotomy for intraesophageal fishbone removal can be summarized into five themes: assumptions during the event, helplessness and dependence before surgery, feelings of fatigue after surgery, sense of benefit during the recovery period, and gratitude and hope.

**Conclusion:**

It is imperative for healthcare professionals to prioritize the psychological rehabilitation of patients by implementing personalized interventions. This approach should aim to facilitate both physical and mental recovery, thereby fostering a renewed sense of hope and well-being in patients.

**Supplementary Information:**

The online version contains supplementary material available at 10.1186/s12876-025-04421-w.

## Introduction

Esophageal foreign bodies represent a frequent clinical emergency [1, 2], necessitating prompt intervention to prevent severe or life-threatening complications, including esophageal ulcers, perforations, penetration into the aorta or pulmonary artery, mediastinal abscesses, and esophagotracheal fistulas [3–5]. Approximately 1,500 people in the United States die annually due to foreign body ingestion [6]. In adults, fish bones, excluding those lodged in the throat, are the most prevalent type of esophageal foreign body(60%) [7]. Their sharp nature markedly elevates the risk of esophageal perforation [8, 9]. Overall, 17.8% of patients had a complication related to the impacted esophageal foreign bodies or to the endoscopic manoeuvers. A surgical approach was required in 3.4% of patients. The overall mortality was 0.85% [10]. Esophageal fish bone impaction is a non-organic condition frequently encountered by individuals, often recurrently, which can lead to delays in diagnosis and timely treatment due to insufficient attention [11]. This delay may result in economic losses and psychological trauma. In clinical practice, the psychological status of patients with intraesophageal fishbone is frequently neglected [10]. However, psychological factors significantly influence the onset, progression, and recovery of this condition [10]. Individuals with intraesophageal fishbone often endure pain, dysphagia, apprehension regarding surgical intervention, and anxiety about potential complications, which can culminate in psychological disturbances such as anxiety and depression [12]. Furthermore, given its classification as a non-organic disorder, the psychological ramifications associated with intraesophageal fishbone warrant careful consideration [10]. Patients may experience psychological trauma as a result of recurrent events, which can have a significant impact on their daily functioning and occupational performance.

Qualitative research is an especially appropriate methodological approach for investigating and comprehending the subjective experiences and emotions of individual patients [13–15]. By thoroughly comprehending the psychological experiences of patients, healthcare professionals can implement psychological interventions more effectively, thereby enhancing patients’ ability to cope with the challenges of their illness and promoting the recovery of both their physical and mental health. Currently, there is a lack of research specifically addressing the psychological experiences of patients with esophageal fish bone impaction. Although esophageal intraesophageal fishbone are a common clinical issue, existing studies have primarily focused on treatment methods and complications, with insufficient attention paid to the psychological experiences of patients. This research aim to fill this gap by conducting both qualitative study to explore patients’ psychological states during diagnosis, treatment, and recovery, providing a scientific basis for clinical psychological interventions.

## Objects and methods

### Study subjects

Between May 2022 and May 2023, the Department of Thoracic Surgery at Tongji Hospital, Tongji Medical College, Huazhong University of Science and Technology, Wuhan, China managed 13 cases of esophageal foreign bodies caused by fish bones, excluding instances where the intraesophageal fishbone were lodged in the throat. Of these patients, 12 were successfully discharged following recovery, whereas 1 patient succumbed to complications. Employing a purposive sampling approach, we conducted comprehensive interviews with the 12 patients who were discharged, with all interviews completed within the five days preceding their discharge.

Inclusion Criteria: The surgical procedure involved a thoracotomy performed under general anesthesia for the removal of intraesophageal fishbone and subsequent esophageal repair. Patients successfully navigated the perioperative period without complications. Eligible participants were required to have the ability to verbally articulate their genuine emotions, possess normal memory function, and exhibit clear cognitive processing, with no history of psychiatric disorders or intellectual/cognitive impairments. Additionally, participants must have engaged in and completed personalized interventions for esophageal fish bone foreign bodies within our department. A willingness to participate in the study and share their experiences was also necessary for inclusion.

Exclusion Criteria: Participants presenting with malignant tumors or severe physical illnesses affecting other anatomical regions were excluded. Additionally, individuals experiencing sudden instability or rapid deterioration of their condition, as well as those who are deceased, were not considered. The demographic and baseline characteristics of the study participants are detailed in Table 1.

**Table 1:** General information of the research subjects

Research Subject	Age range	Occupation	Education Level	Experience with Fish Bone
A	51 ~ 60	self-employed	High School	no
B	31 ~ 40	housewife	College	yes
C	61 ~ 70	Farmer	Primary School	no
D	41 ~ 50	driver	College	no
E	41 ~ 50	Farmer	Junior High	yes
F	61 ~ 70	retiree	High School	no
G	51 ~ 60	Farmer	Junior High	no
H	41 ~ 50	Farmer	Primary School	no
I	51 ~ 60	self-employed	Junior High	yes
J	41 ~ 50	teacher	College	no
K	51 ~ 60	Farmer	Junior High	no
L	51 ~ 60	housewife	Junior High	yes

### Methods

This study is grounded in the phenomenological approach, which focuses on understanding the lived experiences of individuals. We employed Colaizzi’s seven-step method to systematically analyze the qualitative data, ensuring a rigorous and comprehensive exploration of the patients’ psychological experiences.

Additionally, the study incorporates elements of the knowledge-belief-action model, which emphasizes the importance of knowledge in shaping health-related beliefs and behaviors. This framework helps explain how patients’ understanding of their condition influences their psychological responses and coping mechanisms.

#### Interview preparation

The preparation for the interviews involved assembling a recording device, an interview outline, writing instruments, notebooks, and a comprehensive survey questionnaire that collected demographic information such as age, gender, occupation, and educational level.

#### Interview setting

A small, quiet classroom was selected as the interview setting to ensure an environment free from disturbances, with adequate air circulation, optimal temperature, and gentle lighting.

#### Interview scheduling

 Interviews were arranged at mutually convenient times for both the interviewer and the interviewee. The interviewer, who worked in the same department where the data was collected, was available 45 min post-work. Participants were scheduled for interviews outside of their daily activities and treatment procedures. Each participant engaged in two interview sessions, with each session lasting between 30 and 40 min.

#### Interviewer qualifications

The interviewers possessed extensive knowledge regarding esophageal fish bone impaction and had undergone formal training in communication and technical skills. They held at least a secondary-level qualification as psychological counselors and demonstrated empathetic qualities. Additionally, they were adept at engaging with patients using a gentle tone, refraining from subjective assumptions, and effectively establishing a trust-based relationship.

#### Informed consent principle

Prior to conducting the interviews, the interviewer provided a self-introduction and detailed explanation of the research objectives, content, methodology, and anticipated outcomes to the participants. This study received approval from the hospital’s ethics committee, ensuring adherence to the principles of informed consent and confidentiality. To protect the privacy of the participants, pseudonyms were used in place of their actual names in all research materials and publications. Video recordings of the interviews commenced only after obtaining informed consent, and all recordings and notes were destroyed upon the study’s conclusion. The entire qualitative research process was rigorously conducted in accordance with ethical standards.

#### Data collection

This study utilized the Husserlian approach within phenomenological research, with the objective of describing experiences without interpretation, thereby concentrating on an accurate depiction of the real world [16]. In alignment with the research objectives and aims, a comprehensive review of pertinent literature was conducted, and interview guides were formulated in collaboration with subject matter experts [17–19]. The interviews were structured around the following key questions: (1) Can you describe the circumstances that led to the fish bone becoming lodged? (2) What actions did you take in response to the incident? (3) What were your thoughts and feelings upon discovering that surgical intervention was necessary? (4) What insights or lessons have you derived from this experience? (5) What are your plans for your future life? Data collection was carried out via face-to-face, semi-structured, in-depth interviews, facilitated by a team of two interviewers. A total of 24 interviews were conducted (two sessions per participant), with an average duration of 35 min per session. One interviewer was tasked with conducting the interview and managing the audio recording, while the other focused on note-taking and observing the interviewee’s emotional responses, micro-expressions, body language, and tonal variations. Following the completion of the interviews, the audio recordings were transcribed. Interviewees were subsequently informed of the schedule for a follow-up interview and were provided with preliminary findings for validation purposes. The interview guide was reviewed by a panel of experts in the field of psychological research to ensure its validity and relevance.

#### Data analysis

Within 24 h following the interviews, the recorded video data were transcribed into text. The data analysis was conducted using MAXQDA software (version 12.3.5), applying Colaizzi’s seven-step phenomenological analysis method [20, 21]: (1) thoroughly familiarize oneself with the data; (2) identify significant statements; (3) code meaningful perspectives; (4) develop preliminary themes; (5) describe these preliminary themes; (6) formulate a concise and comprehensive phrase for each theme; and (7) seek validation from the interviewees. MAXQDA was used to manage and organize the qualitative data. The software facilitated the coding process by allowing us to systematically categorize and retrieve coded data. It also helped in identifying patterns and relationships within the data, which were crucial for developing the final themes. The software’s features, such as memo functions and visualization tools, were utilized to enhance the rigor and transparency of the analysis process. Both interviewers, who were also the primary researchers, participated in the data analysis process. Two experienced researchers were involved in the coding process. Both coders had extensive training in qualitative research methods and were familiar with Colaizzi’s seven-step method. To ensure consistency and reliability, the following steps were taken: Initial coding was performed independently by both coders. Coders met regularly to discuss and reconcile any discrepancies. A third expert was consulted to resolve any remaining disagreements. The final themes were validated through member checking with participants to ensure accuracy and relevance. Data saturation was achieved through iterative interviews and analysis. The interviews continued until no new themes or insights emerged from the data. Specifically, the criteria for determining data saturation included: Repetition of themes and sub-themes across multiple interviews. Consistency in the descriptions and experiences shared by participants. Absence of new information or perspectives after a set number of interviews (in this case, after 12 interviews).

#### Quality control

The interview outline was meticulously crafted by the interviewers, who aligned it with the research objectives through an extensive review of pertinent literature and consultations with three nursing experts. At the commencement of each interview, the study’s purpose and significance were clearly articulated to the participants, ensuring their comprehension of its importance and fostering an environment conducive to the candid expression of their genuine sentiments. Throughout the interviews, the interviewers posed follow-up questions to clarify ambiguous statements and adeptly probed for insightful responses, all while maintaining a neutral stance and avoiding any form of suggestion or intervention. Post-interview, both the interviewers and participants engaged in reflective analysis of the discussions to ensure the accuracy and depth of the data collected. This process helped in validating the interview content and ensuring that the participants’ perspectives were accurately captured.

The Consolidated criteria for reporting qualitative studies (COREQ): 32-item checklist [17] was used in this study to ensure the quality of the study, the complete COREQ checklistis presented in Table S1.

## Results

The psychological experiences of 12 patients undergoing thoracotomy for esophageal foreign body removal at different stages can be summarized into five themes: assumptions during the event, helplessness and dependence before surgery, feelings of fatigue after surgery, sense of benefit during the recovery period, and gratitude and hope.

### Assumptions during the event

Assumptions pertain to decisions derived from conjecture, encompassing subjective judgment and inference. When individuals experience a fish bone becoming lodged, they frequently attribute their situation to chance, thereby neglecting to adequately address or acknowledge the potential repercussions, which in turn fosters these assumptions. Suboptimal dietary practices may exacerbate the likelihood of such occurrences. For instance, individual A remarked, “Perhaps I ingested the bone while consuming fish soup. It is not uncommon for a bone to become lodged; I assumed it would be harmless to simply swallow it.” Similarly, individual G stated, “I tend to eat rapidly, ingesting food and fish simultaneously, under the belief that I could either expel it through coughing or safely swallow it again without complications.”

### Helplessness and dependence before surgery

Following the lodgment of a fish bone, patients frequently assume that medical intervention is unnecessary. When self-removal attempts prove unsuccessful, they experience confusion and helplessness, eventually seeking hospital treatment. Such feelings of helplessness and passivity contribute to a reliance on healthcare professionals.

* C:* “It’s common to swallow small fish bones while eating fish… this time, it almost cost me my life; what kind of situation is this?”

*I:* “Getting a fish bone stuck is a very ordinary thing. I couldn’t get it out even after eating bread and vinegar, and now I have to undergo surgery; this fish bone is really expensive.”

*H:* “The bone got lodged in a difficult position, so I had to have surgery; I just had to follow the doctor’s orders.”

*K:* “The doctor said the bone was in a dangerous position; I felt it was right to listen to the doctor.” Patients express hope in their doctors, indicating that their helplessness and dependence on medical professionals influence each other.

### Feelings of fatigue after surgery

Fatigue may result from physiological, psychological, and diverse underlying factors, manifesting at any stage of illness. Furthermore, it is frequently a subjective symptom that is overlooked.

#### Physical fatigue

Patients’experiences of physical fatigue are not significantly different from those after other surgeries, which is why healthcare providers may not give it sufficient attention.

(1) Weakness in limbs and coughing is most common.

*A:* “I have no strength to cough or move.”

*K:* “I just want to lie down; I don’t want to move.”

(2) A feeling of heaviness.

*C:* “I have tubes everywhere; moving takes forever.”

*I:* “My body feels like it’s filled with lead; I really try hard, but sitting up is still difficult.”

#### Mental fatigue

Patients experience varying degrees of negative mental and cognitive states.

(1) Feelings of low mood are common.

*B:* “I don’t want to talk to anyone; I feel so tired and don’t want to engage with anything.”

*E:* “I don’t want to do anything.”

*G:* “I feel lethargic; my daughter says she came, but I didn’t even feel that happy.”

(2) Anxiety and insomnia. Patients experience varying degrees of anxiety, which affects their sleep quality.

*A:* “What will happen next? Can I eat again…? I can’t sleep at night and wake up very early in the morning.”

*F:* “Will there be any aftereffects? My body feels exhausted, but I still can’t sleep; I’m afraid to move, worried about pulling on the tubes.”

(3) Dullness. Patients respond slowly to daily activities or care procedures.

* J:* “Once my son asked me if I needed to go to the bathroom, and I didn’t respond for a while; when I saw him with the urinal, I finally understood. (shakes head and smiles)”.

*K:* “The nurse told me to change the nutrition solution and said to pour the milk slowly to avoid bloating; I was a bit dazed and didn’t understand her. (The patient cannot eat orally and requires enteral nutrition.)”.

(4) Lack of concentration.

*D:* “I can’t focus on the TV or when talking to others; I keep thinking about other things.”

### Sense of benefit during the recovery period

The sense of benefit refers to the cognitive and behavioral perception of personal, social, psychological, and spiritual advantages gained from negative life events such as illness or injury. Providing patients with personalized interventions can systematically alleviate postoperative fatigue, leading to positive cognitive and emotional outcomes.

(1) Enhanced awareness of healthy living.

*B:* “I have to take care of the kids, and with late-night studies, I often get angry. I eat quickly… This experience made me realize I need to adjust my schedule and emotions and eat slowly and mindfully.”

*J:* “I need to exercise to boost my immunity; no talking while eating or sleeping.”

(2) Increased engagement in positive living behaviors.

*H:* “I can eat now, and I pay special attention to the rehabilitation training the nurses play; my daughter has already downloaded it to continue at home.”

*L:* “I didn’t expect getting a fish bone stuck would lead to surgery. We’ve set family rules: no talking while eating… I plan to join square dancing after discharge.”

### Gratitude and hope

Research shows that gratitude can reduce physical discomfort and enhance individual psychological resilience, becoming an important factor in promoting health and well-being. Patients feel a sense of happiness from the meticulous care provided by family members and healthcare staff, considering themselves lucky and hoping for a healthy future.

*F:* “Seeing how hard my spouse works to take care of me makes me feel for him; I hope we can live safely together in the future.”

*K:* “We really need to pay attention to our health these days; I hope there won’t be any more accidents… Thank you for teaching me to exercise and sharing your knowledge.”

## Discussion

### Enhancing residents’ dietary practices and awareness of esophageal fish bone impaction

The suboptimal dietary habits of patients increase their susceptibility to incidents of fish bone impaction during fish consumption. Upon inadvertently ingesting a fish bone, patients frequently demonstrate hesitation in seeking medical intervention and may engage in assumptive reasoning. Failure to promptly seek hospital treatment, opting instead for home remedies, can result in severe complications due to delayed medical intervention. Participants in this study reported that experiencing a fish bone impaction is a common occurrence and often regarded it as a minor issue that can be easily resolved. However, their understanding of the consequences of esophageal fish bone impaction was lacking, reflecting a deficiency in relevant knowledge. This finding is consistent with previous research by Aiolfi et al. (2018), who reported that patients with esophageal foreign bodies often underestimate the severity of their condition and delay seeking medical help, leading to complications [10]. Similarly, Sung et al. (2011) highlighted that patients’ lack of awareness about the potential risks of esophageal fish bone impaction contributes to their reliance on home remedies, which can exacerbate the problem [8]. These studies collectively emphasize the critical need for targeted health education to improve public awareness and prompt medical intervention.

The knowledge-belief-action model in health education underscores the pivotal role of knowledge as the foundation for developing health-related beliefs and attitudes, which in turn facilitate the adoption of healthy behaviors [22]. Healthcare professionals are encouraged to actively engage in educational outreach initiatives. This can be achieved through the organization of in-person lectures and online courses aimed at disseminating pertinent knowledge, thereby enhancing community awareness. Additionally, they should focus on the distribution of informational pamphlets and the creation and dissemination of promotional videos and illustrations through WeChat public accounts.

Moreover, it is crucial to enhance parents’ capacity to identify the risk of fish bone impaction in children. To increase residents’ adherence to preventive measures, organizing community health check-ups and free clinics could be beneficial. Additionally, incorporating real-life case studies and realistic public service advertisements into promotional materials can effectively communicate the significance of healthy dietary practices and the potential consequences and severity of esophageal fish bone incidents. Utilizing diverse channels and formats can facilitate a broader understanding of these issues, thereby improving patients’ cognitive awareness and encouraging proactive behavior.

### Patient participation in personalized interventions to improve postoperative fatigue

Personalized interventions for patients experiencing esophageal fish bone impaction primarily encompass benefit-finding education alongside routine pulmonary rehabilitation training, which function synergistically [23]. The latter intervention emphasizes the provision of breathing exercises, rehabilitation guidance, and nutritional recovery, aiming to improve pulmonary function, enhance muscle strength, and bolster immune response. Enhancing education on benefit-finding is essential for fostering healthy behaviors and augmenting patient engagement. Presently, the clinical emphasis on psychological care predominantly targets the prevention and reduction of psychological issues, frequently overlooking the extent to which patients can effectively comprehend and assimilate this care [23]. Our findings align with those of Sanjuán et al. (2016), who demonstrated that benefit-finding education can significantly enhance psychological resilience and overall well-being in patients undergoing surgical interventions [23]. Additionally, Willard et al. (2016) reported that integrating positive psychology into patient care can improve recovery outcomes and reduce postoperative fatigue [24]. These studies support our conclusion that personalized interventions, particularly those focused on benefit-finding, can be highly effective in improving patient outcomes. In line with current literature, the importance of perioperative nursing care in facilitating patient recovery and improving postoperative outcomes cannot be overstated.

Benefit-finding education seeks to assist patients in reframing adverse situations positively, thereby enhancing their psychological resilience and coping mechanisms in alignment with their unique personality traits. Through the provision of education grounded in positive psychology, patients are enabled to discern potential benefits, which facilitates improved management of fatigue and augments their self-care capacities. The beneficial impacts of positive psychology can be achieved through approaches such as cognitive behavioral stress management, peer support, and complementary therapies [24, 25]. Healthcare practitioners can employ empathetic understanding and a variety of intervention strategies, utilizing the Benefit Discovery Scale to evaluate patients’ receptivity to and perceived advantages of psychological interventions [26]. When patients cultivate a positive mindset, they are more inclined to participate in pulmonary rehabilitation, thereby attaining both psychological and physiological satisfaction.

The respondents in this study experienced advantages from personalized interventions, which offered novel insights and directions for psychological care, facilitating their ability to confront and accept their circumstances. Through the active pursuit and engagement with pertinent information, patients can be directed towards the cultivation of positive emotions, consequently mitigating fatigue.

### Practical significance of qualitative research on the psychological experiences of patients undergoing esophageal foreign body removal

In recent years, scholarly investigations concerning patients with esophageal fish bone impaction have predominantly concentrated on therapeutic interventions [10]. Conversely, nursing research has largely been confined to individual case studies, analyses of comorbid conditions, and experiential accounts. The restricted sample size and temporal scope associated with this patient population have constrained the capacity for quantitative analysis and comprehensive research. This study endeavors to address this gap by eliciting authentic psychological experiences from patients at various stages through the method of in-depth interviews. Research indicates that when healthcare providers comprehend the distinct personality traits of patients and cultivate trust through empathetic interactions, patients are more inclined to disclose their innermost feelings [13, 27]. Upon expressing their emotions and perceiving acceptance, patients tend to experience a sense of relaxation both physically and mentally. Our qualitative approach is supported by similar studies that highlight the importance of understanding patients’ subjective experiences to improve care. For instance, Willemsen (2022) emphasized the value of qualitative case studies in providing deeper insights into patient psychology, which can inform more personalized and effective interventions [13]. Additionally, Bergdahl and Berterö (2015) argued that qualitative research can reveal aspects of patient care that quantitative studies might overlook, such as the emotional and psychological dimensions of recovery [15]. These studies collectively underscore the importance of qualitative research in enhancing patient-centered care.

Furthermore, the study demonstrated that personalized interventions not only mitigate postoperative fatigue but also that the timing of these interventions may play a crucial role in its prevention. Consequently, healthcare providers should be attentive to both the physical and psychological changes experienced by patients to prevent complications and emotional distress associated with prolonged fatigue, which may arise in the absence of timely intervention.

While this study provides valuable insights into the psychological experiences of patients undergoing thoracotomy for esophageal foreign body removal, several limitations should be acknowledged. First, the sample size is relatively small and limited to a specific geographic region, which may affect the generalizability of the findings. Future research should consider larger and more diverse samples to enhance the applicability of the results. Second, the study relies on self-reported data, which may be subject to recall bias and social desirability bias. Incorporating objective measures, such as physiological assessments or third-party observations, could provide a more comprehensive understanding of patients’ experiences. Third, the study focuses on the immediate postoperative period and short-term recovery, and further research is needed to explore the long-term psychological and functional outcomes of patients. Finally, the study’s qualitative design, while rich in detail, does not allow for statistical generalization. Future studies could benefit from a mixed-methods approach, combining qualitative insights with quantitative data to provide a more robust analysis.

## Conclusion

This study utilized phenomenological analysis to ascertain that dietary habits and awareness are significant contributors to incidents of esophageal fish bone impaction. Personalized interventions have been identified as effective strategies for mitigating postoperative fatigue in patients. Esophageal fish bone impaction not only incurs economic losses and psychological trauma for patients but also substantially impacts their long-term quality of life. Consequently, it is imperative to promote proactive measures among community members to prevent incidents of esophageal fish bone impaction and to cultivate awareness regarding the appropriate management of such occurrences. Healthcare professionals should be trained to employ empathetic understanding in delivering timely and appropriate personalized interventions to address the psychological concerns of patients. This study seeks to contribute a reference framework for future psychological care and health interventions targeting individuals experiencing esophageal fish bone impaction.

## Supplementary Information

Supplementary Material 1

## Data Availability

The datasets used and analysed during the current study are available from the corresponding author on reasonable request.
