# Supplementary material for: A qualitative investigation into the psychological experiences of patients undergoing thoracotomy for the removal of intraesophageal fishbone
Source: BMC Gastroenterol. 2025 Nov 18;25:815. doi: 10.1186/s12876-025-04421-w (PMC12625274; doi:10.1186/s12876-025-04421-w)
Supplement: Supplementary file 1 — Supplementary Material 1 [file 12876_2025_4421_MOESM1_ESM.docx]

Table S1 Consolidated criteria for reporting qualitative studies (COREQ): 32-item checklist

| No Item | Description |
| --- | --- |
| Domain 1: Research team and reflexivity  Personal Characteristics |  |
| 1. Interviewer/facilitator | Author Xiaoying Hou conducted all the interviews for this study. |
| 2. Credentials | Authors Xiaoying Hou and Junhui Wang have undergraduate degrees in nursing, and author Wenbin Zou has an M.D. degree |
| 3. Occupation | Authors Xiaoying Hou and Junhui Wang are nurse practitioners and author Wenbin Zou is an associate physician |
| 4. Gender | Authors Xiaoying Hou and Junhui Wang are Female and author Wenbin Zou is male. |
| 5. Experience and training | The authors have more than 5 years of qualitative interviewing experience in the field of health research and specialized training in semi-structured interviewing and data analysis. |
| 6. Relationship established | No prior relationship was established with participants before the study began. Participants were recruited via local healthcare organizations, which facilitated access to eligible candidates. |
| 7. Participant knowledge of theinterviewer | Participants were informed that the interviewer was a researcher with expertise in behavioral science and qualitative research methods. |
| 8. Interviewer characteristics | The interviewer Xiaoying Hou was aware of potential biases based on her professional background in health behavior. To minimize this, the interviewer adopted a neutral stance and encouraged participants to share their own perspectives freely. |
| Domain 2: study design  Theoretical framework |  |
| 9. Methodological orientation and  Theory | This study used a phenomenological approach to explore participants' experiences of medical care. |
| 10. Sampling | Participants were selected using a purposive sampling method to ensure that patients who had undergone surgical treatment for intraesophageal fish spines were able to participate. |
| 11. Method of approach | Participants were contacted by the research team through face-to-face interviews. |
| 12. Sample size | There were a total of 13 participants in this study. This sample size was determined based on the qualitative nature of the study and was intended to saturate the data. |
| 13. Non-participation | One person was unable to participate in this study due to postoperative death |
| 14. Setting of data collection | We collected data by conducting semi-structured interviews face-to-face. |
| 15. Presence of non-participants | No non-participants were present during the interviews. The interviewer and the participant were the only people involved in the data collection. |
| 16. Description of sample | Twelve patients with open thoracic esophageal fishbone removal were included in this study. |
| 17. Interview guide | The interview guide was developed by the research team based on existing literature and pilot testing. |
| 18. Repeat interviews | No repeat interviews were conducted. |
| 19. Audio/visual recording | All interviews were audio recorded with participants' consent for accuracy and to facilitate transcription. |
| 20. Field notes | Field notes were taken during each interview to document the researcher’s impressions, particularly noting non-verbal cues and emotional reactions. |
| 21. Duration | Interviews lasted between 60 and 90 minutes each. |
| 22. Data saturation | Data saturation was discussed and was determined to be achieved after 12 interviews, when no new themes emerged during the final three interviews. |
| 23. Transcripts returned | Transcripts were returned to participants for comment and correction. Four participants provided feedback on the accuracy of the transcriptions. |
| Domain 3: analysis and findings  Data analysis |  |
| 24. Number of data coders | Two researchers, Xiaoying Hou (the interviewer) and Junhui Wang, a trained qualitative analyst, independently coded the data. |
| 25. Description of the coding tree | The researcher counted through a single level of categorization, but no coding tree description was performed in the text |
| 26. Derivation of themes | Themes were derived directly from the data, rather than being predefined. |
| 27. Software | Managing data with maxqda Chinese version |
| 28. Participant checking | All participants were invited to review and provide feedback on the study results. |
| 29. Quotations presented | Participant quotes are included in the findings to illustrate key themes.  Participants were numbered using letters. |
| 30. Data and findings consistent | The data presented is consistent with the findings, as all major themes were clearly supported by multiple participant quotes and corroborated by the research team’s analysis. |
| 31. Clarity of major themes | The main themes are clearly presented in the findings with ample citations and discussion. |
| 32. Clarity of minor themes | Description of different cases or discussion of sub-themes was not conducted in this study |
